# Tailoring of pore structure in mesoporous carbon for favourable flavin mediated interfacial electron transfer in microbial fuel cells†

**DOI:** 10.1039/c8ra00436f

**Published:** 2018-03-06

**Authors:** Wei Tang, Xiao-Shuai Wu, Yan Qiao, Rui-Jie Wang, Xian Luo

**Affiliations:** Institute for Clean Energy and Advanced Materials, Faculty of Materials and Energy, Southwest University Chongqing 400715 China yanqiao@swu.edu.cn; Chongqing Key Laboratory for Advanced Materials and Technologies of Clean Energies Chongqing 400715 P. R. China

## Abstract

Mesoporous carbon (MC) is supposed to be a good candidate for microbial fuel cell (MFC) anodes as it possesses a large specific area for the redox reaction of the electron shuttles and should deliver high power density. However, the power generation performance of MC anodes is often un-satisfying. It seems that a large portion of the pore surface is not available for anodic redox reaction but the reason is not clear. Here, three MCs with different pore sizes and pore shapes were fabricated and used to explore the effect of the pore structure on the bioelectrocatalysis in *Shewanella putrefaciens* CN32 MFCs. It is interesting that MC with 40–60 nm spheric pores (MC-III) possesses superior bio-electrocatalytic performance to the CMK-3 (MC-I with 3 nm channel like pores) and the one with 14 nm spheric pores (MC-II) although the specific surface area of MC-III is lower than MC-II and MC-I. The reason might be that the MC-III provides a more favorable pore structure than the other two MCs for flavin based redox reaction at the interface between the biofilm and the electrode. As a result, the MC-III anode delivered the highest power density at around 1700 mW m^−2^, which is 1.6 fold higher than that of the MC-I anode. A possible mechanism for the pore shape/size dependent interfacial electron transfer process has also been proposed. This work reveals that spheric mesopores with large pore width could be more favorable than the narrow channel-like pores for flavin based interfacial electron transfer in biofilm anodes, which will provide some insights for the design of the mesoporous anode in MFCs.

## Abstract 2

Spherical mesopores with large pore width are more favorable to flavin mediated interfacial electron transfer in microbial fuel cells.

## Graphical abstract

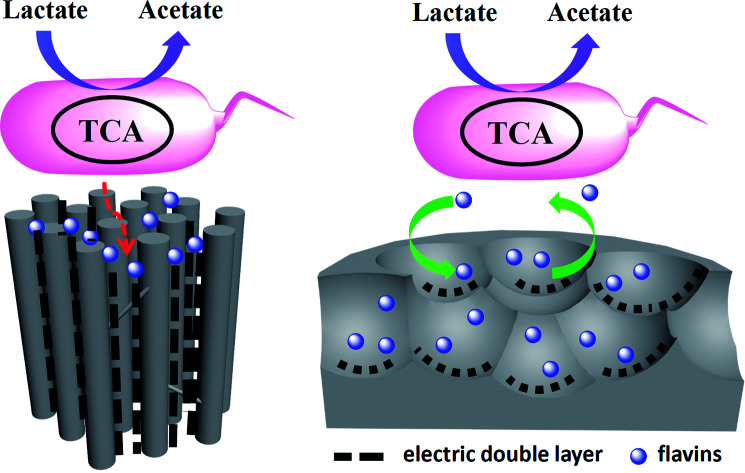

## Introduction

The MFC is a promising technology that can implement wastewater treatment and biomass power generation synchronously.^1–4^ Currently, the low power output of MFCs seriously restricts the development of this technology and practical applications. There are many factors that can affect MFCs' electricity generation performance, such as the battery configuration, ion conductivity of the proton exchange membrane, electrocatalysis in the anode and cathode as well as the electroactivity of the biocatalysts – the microorganisms.^5–9^ It is believed that the electrocatalysis of the bioanode is the most important one, which is determined by the interfacial electron transfer between the exoelectrogens and the electrode.^10^ To promote this process, some researchers have attempted to improve the electrocatalytic activity of the bacteria cells by gene manipulation^11,12^ but more works were devoted into the fabrication of anode materials.^13–15^

As the anodic electrocatalysis relies on the biocatalysts loading, porous materials especially the porous carbon materials are good candidates for high performance anodes.^16,17^ At the same time, the present of mesopores on the pore walls of macroporous materials could promote the diffusion of nutrition as well as the redox reaction of electron shuttles. For example, a three-dimensional hierarchically nanostructured carbon anode with well-patterned macropores (∼400 nm) and ordered mesopores (∼4 nm) has delivered high power density in an anaerobic sludge MFC.^18^ The reduced graphene oxide aerogels with mesopores also exhibit good bioelectrocatalytic performance in *Shewanella putrefaciens* CN32 MFCs^19,20^ although the mechanism of the interaction between graphene and the biofilm is not very clear.^21^ Although various porous carbon materials have been applied as anode materials for MFCs, few reports focus on the effect of pore structure or pore size on the bio-electrocatalysis, especially for the interfacial electron transfer in the anodes.

For different kinds of exoelectrogens, the self-excreted electron shuttles play crucial roles in the interfacial electron transfer between bacteria cells and the electrode surface.^22^ Flavins, including flavin mononucleotide (FMN) and riboflavin, are self-generated electron shuttles of Shewanella species,^23^ which not only mediate the indirect extracellular electron transfer but also take part in the direct electron transfer process.^24^ It has been reported that the increase of pore size from micropores to mesopores could promote the flavin based electron transfer by allowing the two-electroactive sites simultaneously access the electrode surface.^25^ A mesoporous structured anode with appropriate pore size or pore shape could greatly improve the output performance of the MFCs. However, it is still unclear that which kind of mesopores would be favorable for flavin based electron transfer.

In this work, we fabricated three kinds of mesoporous carbon with different pore size to investigate their performance in flavin based extracellular electron transfer. The relationship between the pore structure and the anodic electrocatalysis were deeply discussed and a possible mechanism was proposed.

## Experimental

### Materials

Sucrose (Beijing Dingguo Changsheng Biotechnology), boric acid (Sigma-Aldrich), silicon(iv) oxide, 15% in H_2_O colloidal dispersion, (Alfa Aesar), LudoxHS40 (Sigma Aldrich), Polytetrafluoroethylene (PTFE) solution (1 wt%) (Sigma-Aldrich) were used in the present study. *Shewanella putrefaciens* CN32, a dissimilatory metal-reducing bacterium was purchased from ATCC (number: BAA-1097). Deionized (DI) water of resistance 18.2 MU was used for all experiments.

### Preparation of MCs

The MC-I was the commercial CMK-3 that provided by Nanjing XFNANO Materials. The MC-II and MC-III were prepared using the nanocasting method.^26^ In the process of synthesis, sucrose was used as the carbon precursor and colloidal silica with different particle sizes as the hard template and boric acid as the pore expanding agent to reach the various pore sizes of carbon materials. An example of synthetic MC materials, we added 13.72 g of 4 nm colloidal silica solution to sucrose (6.25 g), sulfuric acid (0.71 g), H_2_O (50 mL), and boric acid solutions (5.65 g). For a start, the mixtures were dried at 140 °C in an oil bath pan with silicone oil. Next, the brown powder was carbonized at 900 °C with N_2_ flow with a heating rate of 5 °C min^−1^ for 3 h. Then hydrofluoric acid was etching carbon powder to remove silica and boron species.

### Bacteria growth

*S. putrefaciens* CN32 was grown anaerobically in 5 mL of Luriae Bertani (LB) broth medium overnight, which was a mixture of 10 g L^−1^ sodium chloride, 10 g L^−1^ tryptone, 5 g L^−1^ yeast extract. Drawing 1 mL of bacterial culture suspension inoculate in 100 mL of fresh LB broth, and then the bacterial culture suspension was cultivated with shaking at 30 °C until the optical density at 600 nm (OD600) reached about 1.0–1.5. The cells were harvested by centrifugation at 5000*g* for 5 min and washed 3 times with fresh M9 buffer (Na_2_HPO_4_, 6 g L^−1^; KH_2_PO_4_, 3 g L^−1^; NaCl, 0.5 g L^−1^; NH_4_Cl, 1 g L^−1^; MgSO_4_, 1 mM; CaCl_2_, 0.1 mM). Subsequently, the cell pellets were re-suspended in 100 mL fresh electrolyte (M9 buffer supplemented with 18 mM lactate as electron donor) and then transferred into the anodic chamber of MFCs followed by purged with nitrogen gas to remove the dissolved oxygen.

### MFC construction and system set-up

The dual-chamber MFCs reactors were constructed using two 100 mL glass flasks. Each chamber had an around 70 mL liquid volume of liquid and the separation of two chambers was used by a proton exchange membrane (PEM, Nafion 117, DuPont Del.). The MC/carbon cloth with loading amount of 5 mg cm^−2^ was used as anode and the carbon brush was used as cathode. The cathodic electrolyte was 50 mM K_3_[Fe(CN)_6_] in 0.1 M PBS (pH 7.4) in order to obtain comparable data with our previous work. The external resistance for the constant load discharge experiments was 1.5 kΩ.

### Physical characterization

The Brunauer–Emmett–Teller (BET) surfaces areas and porosity of the samples were evaluated by nitrogen sorption isotherms measured at 77 K using an automatic adsorption instrument NOVA 1200e (Quantachrome, Boynton Beach, Florida). After discharge, the surface morphologies of anodes were studied with a field emission scanning electron microscope (FESEM, JEOL7800F). Before morphology observation, the biofilm adhered anodes (cut from the anodes) were fixed in 4% polyoxymethylene solution for more than 12 h to stabilize the bacteria attached to the anodes. Then the samples were sequentially dehydrated in ethanol series (30%, 40%, 50%, 60%, 70%, 80%, 90%, and 100%) and dried in vacuum at room temperature overnight.

### Electrochemical measurements

All electrochemical experiments were carried out with CHI 660E electrochemical working station (CHI Instrument, Shanghai, China) in a three-electrode cell including the working electrode, a saturated calomel (sat. KCl) reference electrode and a titanium plate as counter electrode. Unless otherwise stated, electrochemical impedance measurements (EIS) for the MCs electrodes were performed over a frequency range of 0.01 Hz to 100 kHz at −0.45 V (*vs.* SCE). All the electrochemical measurements were conducted at room temperature.

## Results & discussion

**Fig. 1:**
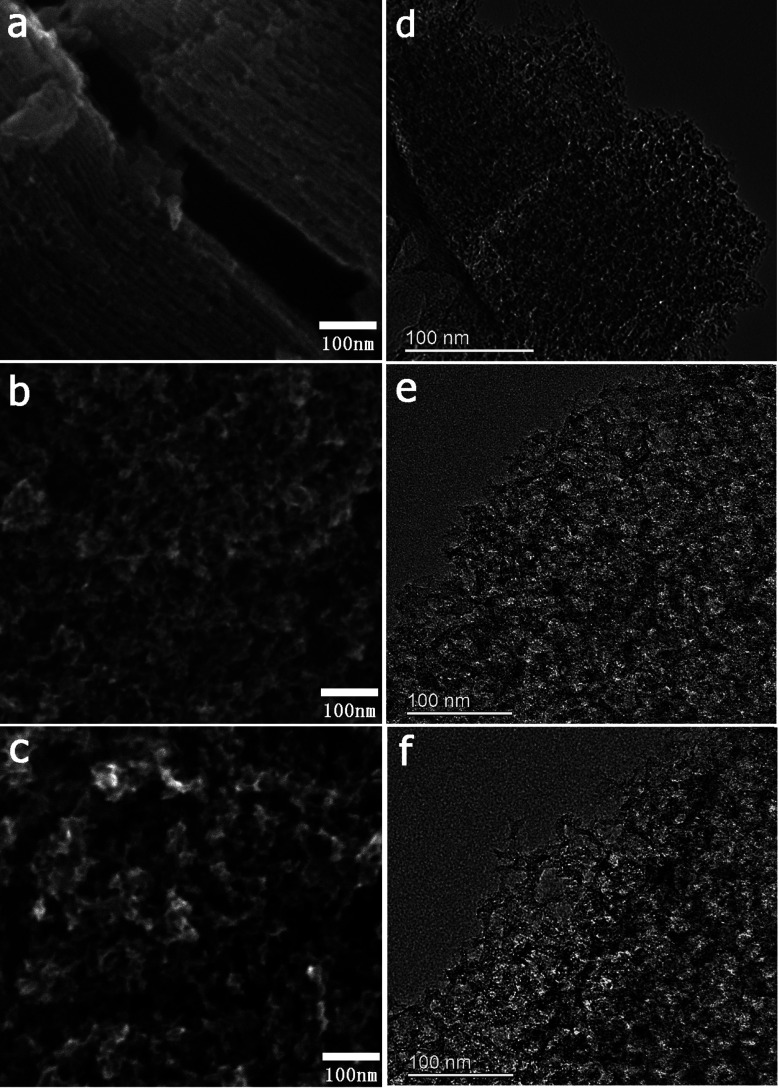
SEM (a–c) and TEM (d–f) images of MCs. (a and d) MC-I; (b and e) MC-II; (c and f) MC-III.

**Fig. 2:**
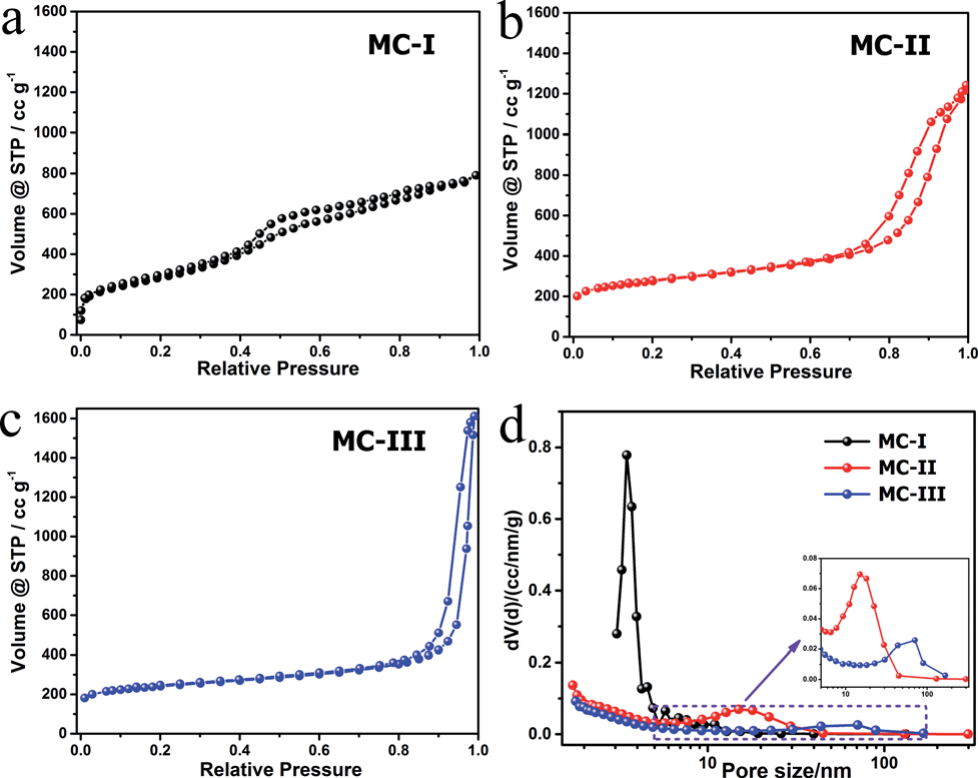
Isotherm plots (a–c) and the pore size distribution curves (d) of the MCs.

The FESEM and TEM images of the MCs are shown in Fig. 1. The MC-I has a typical hexagonally ordered mesostructured, which constructed with hexagonal pattern of carbon rods.^23^ For MC-II and MC-III, they show spherical mesopores that are replicated from the silica spheres. It is noted that the pore size is increased as the order of MC-I, MC-II and MC-III, which can be further proved by the pore structure analysis results. The isotherms of the MCs (Fig. 2) show that the isotherm of MC-I has a typical type IV nitrogen adsorption isotherm with H_2_ hysteresis loop, which indicates an ink-bottle pore shape. While the MC-II exhibit type IV nitrogen adsorption isotherm with a broad intermediate hysteresis loop between H1 and H2, which indicates uniform size cylindrical pores or agglomerates of spheroidal particles with uniform size. For MC-II, a capillary condensation step occurred at a relative pressure that ranged from 0.65 to 0.9. This step shifted to higher relative pressure in MC-III. It is suggests that the pore size of the MCs increases gradually from MC-II to MC-III. This is in accordance with the results of pore size distribution (Fig. 2d). The MC-I has a narrow pore size distribution at about 3 nm while the MC-II and MC-III have primary pore size of about 15 nm and 60 nm respectively, which is in agreement with SEM and TEM micrographs. Accordingly, the MC-I has higher BET surface area (1031.15 m^2^ g^−1^) than that of MC-II (901.05 m^2^ g^−1^) and MC-III (773.61 m^2^ g^−1^). The surface chemistry properties of the MCs were also investigated with XPS. The results (Fig. S1†) show that all of these three MCs have similar surface properties as the total XPS spectra are almost same. Therefore, the effect of the surface properties on the interfacial electron transfer and the bioelectrocatalysis could be neglected.

**Fig. 3:**
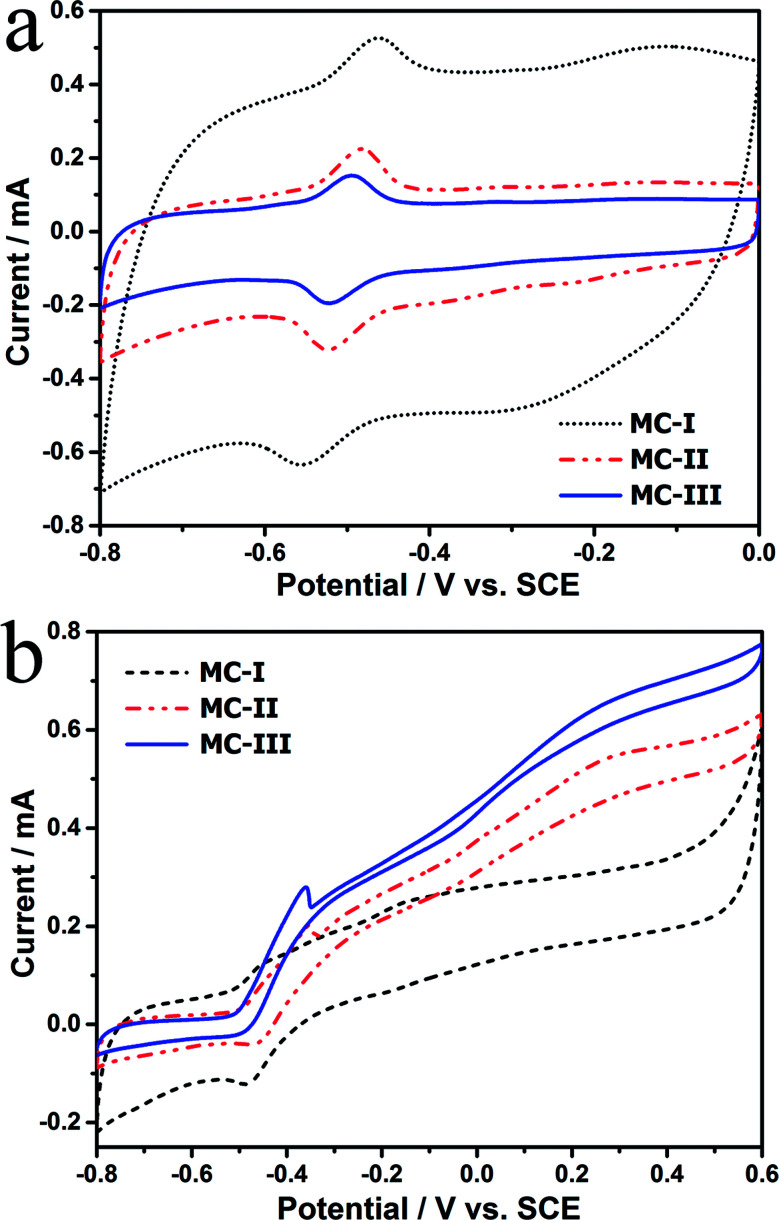
(a) CVs of different MC anodes in phosphate buffer with 5 μM FMN. (Scan rate is 5 mV s^−1^) (b) CVs with turnover current in anaerobic M9 buffer with *S. putrefaciens* CN32 cell suspension. (Scan rate is 1 mV s^−1^).

To explore the effect of pore structure on the electrocatalytic activity, the electrochemical behavior of different MC electrodes were investigated in 0.1 M phosphate buffer with 5 μM flavin mononucleotide (FMN) at first. From the cyclic voltammograms (Fig. 3a), all of the three MCs show a pair of well defined redox peaks for FMN redox reaction. The MC-I has much higher capacitive current than MC-II and MC-III, which might be due to its large specific surface area. To clearly present the redox peaks of FMN, the baseline subtracted CVs are shown in Fig. S2.† It is interesting that the peak current of MC-I is not higher than that of MC-II, which suggests that the part of the surface of MC-I is not accessible for FMN redox reaction. It is also noted that the MC-II and MC-III have much smaller peak separation than that of MC-I. That means the redox reaction of FMN is much faster on the surface of MC-II or MC-III than that on MC-I, which is in accordance with the results from EIS analysis. From the Nyquist plots (Fig. S3†) and the simulated calculation, the charge transfer resistances for OMC-I, OMC-II and OMC-III are 21.4 ohms, 22.3 ohms and 27.1 ohms respectively. It is possible that the large spherical pores are more suitable for flavin redox reaction than the narrow channel-like pores.

Further, the electrocatalytic behaviour of the biofilm adhered MC anodes were investigated in anaerobic M9 buffer with *S. putrefaciens* CN32 cell suspension. According to the baseline subtracted CVs (Fig. S4†) the MC-II and MC-III shows similar redox behaviour as that in FMN solution with a little bit wider peak separation, which could be due to the decreased ion strength in the M9 buffer. However, the MC-I anode presents a poor kinetic process with much lower peak height and much wider peak separation. This result suggests that the redox reaction of flavins on the MC-I surface is partially blocked. According to the CVs obtained at different time (data not shown), the peak current increases with the discharging time for both MC-II and MC-III anodes. While for MC-I the increment in peak current is much lower. It has been demonstrated that the decreased ionic strength could increases the thickness of electric double layer, which will narrow down the diameter of mesopores and block the diffusion of bulk solution.^27^ In this case, the low ionic strength of anolyte could be the reason for the poor electrochemical behavior of MC-I anode. It is also noted that the MC-III possesses faster charge transfer than that of MC-II when measured in anolyte. Therefore, the large mesopores could overcome the impediment of the thick electric double layer on the electrolyte steeping process and thus more favourable for the flavin redox reaction. In the meantime, the CVs with turn over current were also measured at slow scan rate (1 mV s^−1^, Fig. 3b). The on-set potential for three anodes are almost same (at around −0.45 V *vs.* SCE) but the MC-III has the highest steady state catalytic current (at potential above 0.2 V *vs.* SCE) while the MC-I has the lowest one. This result indicates that the MC-III anode has best electrocatalytic performance, which could be due to the favourable pore structure for flavin based interfacial electron transfer.

**Fig. 4:**
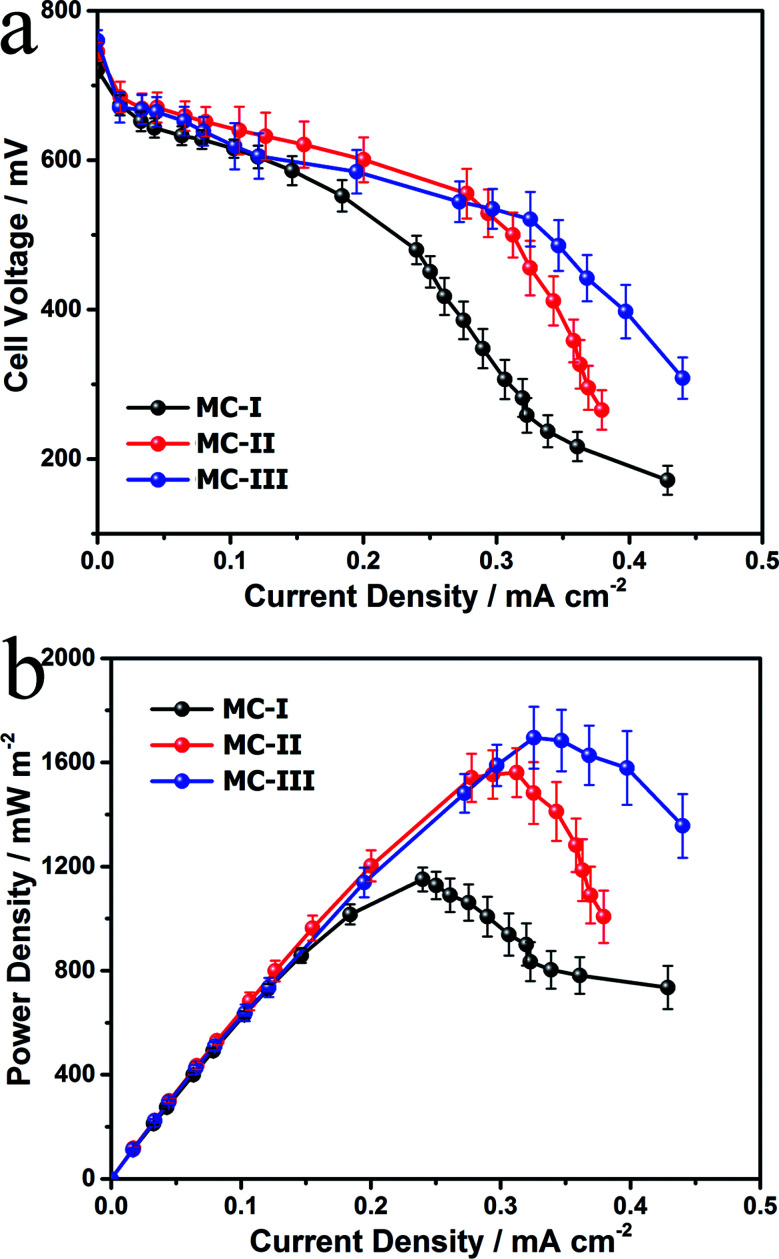
Polarization curves (a) and power curves (b) of the MFCs with different MC anodes.

To evaluate the power generation performance of the MC anodes, power curves and the polarization curves of the MFCs with different MC anodes were examined with a series of external loads. The open circuit voltages for the three MFCs are almost same but the cell voltage of the MFC with MC-I anode drops fast as the increase of current density (Fig. 4a). Consequently, the MFC with MC-III anode delivers maximum power density at around 1700 mW m^−2^, which is higher than the other two MFCs (1150 mW m^−2^ and 1560 mW m^−2^ respectively) and also the previous reported MFCs with polyaniline hybridized large-mesoporous carbon anode,^28^ carbon nanotube anode^29^ or with carbon cloth anode.^30^ After discharge, the surface morphology of the MC anodes were observed by using FESEM. The results (Fig. S5†) show that there are thick biofilm on all of these three anodes, which suggests that, the pore structure variation in these MCs does not affect the biofilm formation on the anode. In this case, the difference in the bioelectrocatalytic performance should be mainly attributed to the flavin based interfacial electron transfer between the biofilm and the electrode.

**Fig. 5:**
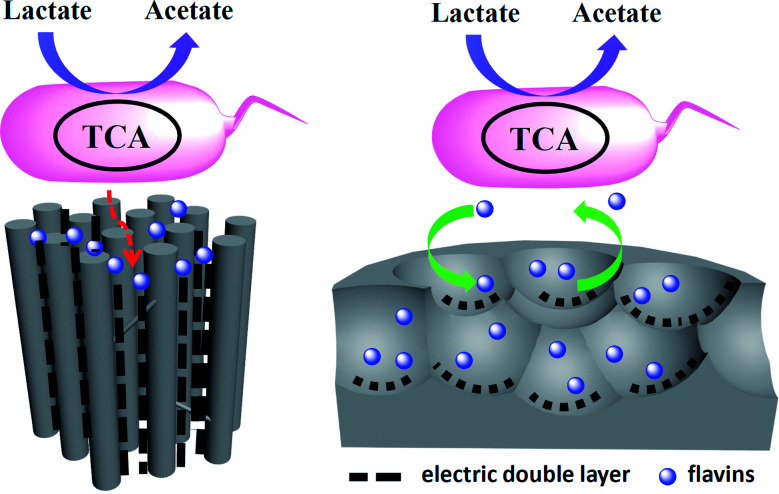
Mechanism diagram of the pore shape/size effect on the flavin based interfacial electron transfer.

According to the above results, a possible mechanism was proposed to explain the pore shape/size effect on the interfacial electron transfer (Fig. 5). Considering the size of the flavins is about 1–1.5 nm at the lowest energy state,^25^ the pores with the pore size over 3 nm should be enough for the flavins diffusion in and the redox reaction. However, the thickness of the electric double layer will also affect the effective area of a mesoporous electrode that available for electrochemical reaction.^27^ According to Gouy–Chapman theory, as electrolyte concentration increases, double-layer thickness decreases.^31^ For the low ionic strength solution like 5 mM phosphate buffer, the double-layer thickness could reach 2–4 nm.^32^ Hence, the MC-I shows reasonable flavin redox behavior when the electrode was put into 0.1 M phosphate buffer. When the MC-I was applied in MFC anode with M9 medium (with similar ionic strength as 30 mM phosphate buffer), the relative low ion strength resulted in the increase of double-layer thickness, which would narrowed down the pore width and block the flavin redox reaction. For MC-II and MC-III, the wider size spherical mesopores could be accessible for flavins so that they can deliver higher faradic current although the specific surface area is lower than that of MC-I. The superior electrocatalytic performance of MC-III to MC-II might be due to the match of the curvature of the pores to the surface roughness of the bacteria cells for direct interfacial electron transfer. It still needs further investigation or calculation to prove.

## Conclusions

In this work, the flavin based interfacial electron transfer process was investigated on three MC electrodes with different pore shape and pore size. The MC-III with large (40–60 nm) spherical pores and MC-II with 14 nm spherical pores show better interfacial redox reaction as well as the bioelectrocatalytic performance than that of MC-I with 3 nm channel like mesopores. An electric double layer involved mechanism was proposed to explain the blocked flavin redox reaction on the MC-I anode. This work suggests that the mesoporous carbon with wide spherical mesopores could be more favourable than the one with the small mesopores for the flavin based interfacial electron transfer since the low ionic strength in MFC anode will increase the electric double layer and decrease the accessible area. This work will provide some insights for the design of the mesoporous anode in MFCs.

## Conflicts of interest

There are no conflicts to declare.

## Supplementary Material

RA-008-C8RA00436F-s001
