# Supplementary material for: Tailoring of pore structure in mesoporous carbon for favourable flavin mediated interfacial electron transfer in microbial fuel cells
Source: RSC Adv. 2018 Mar 6;8(18):9597–602. doi: 10.1039/c8ra00436f (PMC9078639; doi:10.1039/c8ra00436f)
Supplement: RA-008-C8RA00436F-s001 [file RA-008-C8RA00436F-s001.pdf]

Electronic Supplementary Material (ESI) for RSC Advances.
This journal is © The Royal Society of Chemistry 2018

Supporting Information

Tailoring of pore structure in mesoporous carbon for favourable flavin mediated interfacial electron transfer in microbial fuel cells

Wei Tang[ab], Xiao-Shuai Wu[ab], Yan Qiao[ab]*, Rui-Jie Wang[ab], Xian Luo[ab]

a. Institute for Clean Energy and Advanced Materials, Faculty of Materials and Energy, Southwest University, Chongqing 400715, China

b. Chongqing Key Laboratory for Advanced Materials and Technologies of Clean Energies, Chongqing 400715, P.R. China

E-mail: yanqiao@swu.edu.cn..

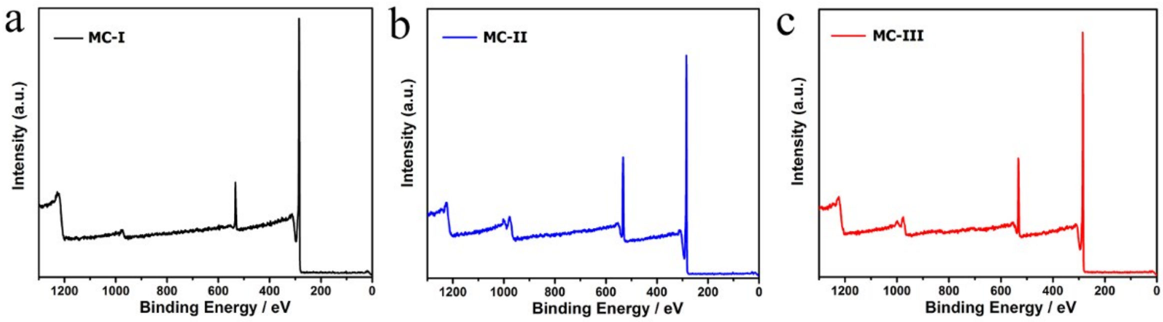

Figure S1. XPS spectra of the OMCs.

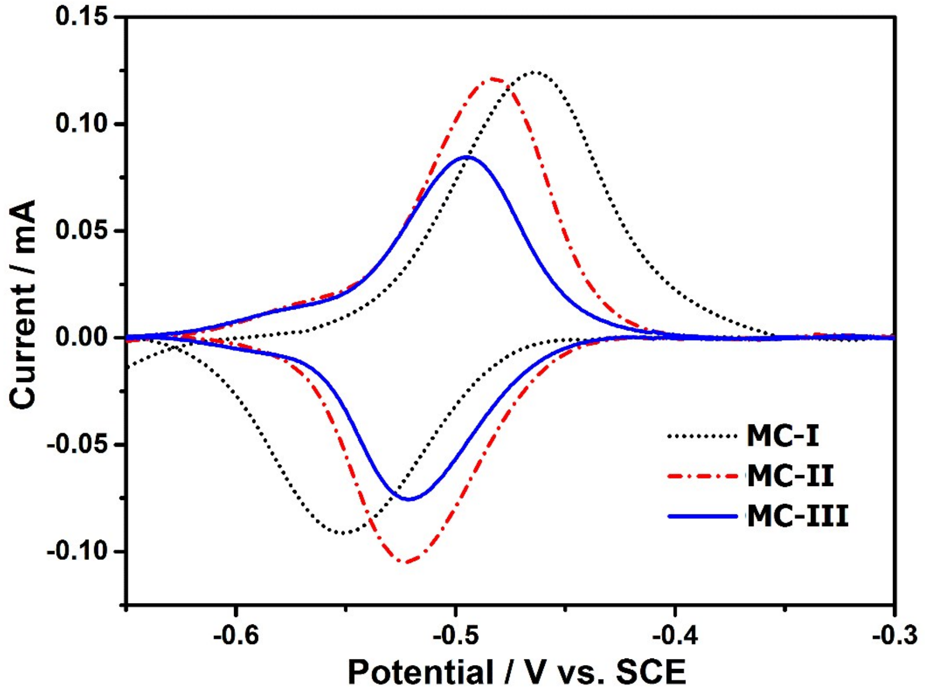

Figure S2. Baseline subtracted CVs of OMC electrodes in phosphate buffer with 5μM FMN. (Scan rate is 5mV s^{-1})

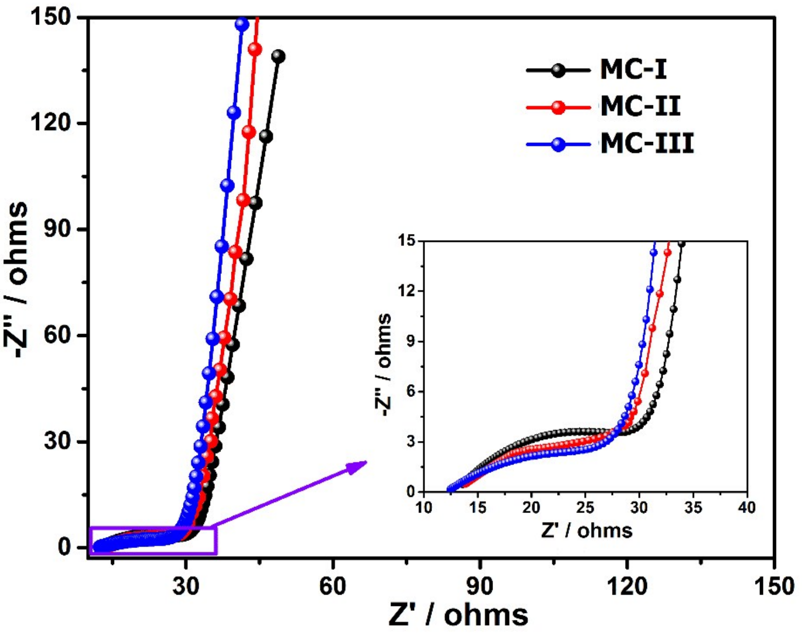

Figure S3. Nyquist plots of OMC electrodes in phosphate buffer with 5μM FMN.

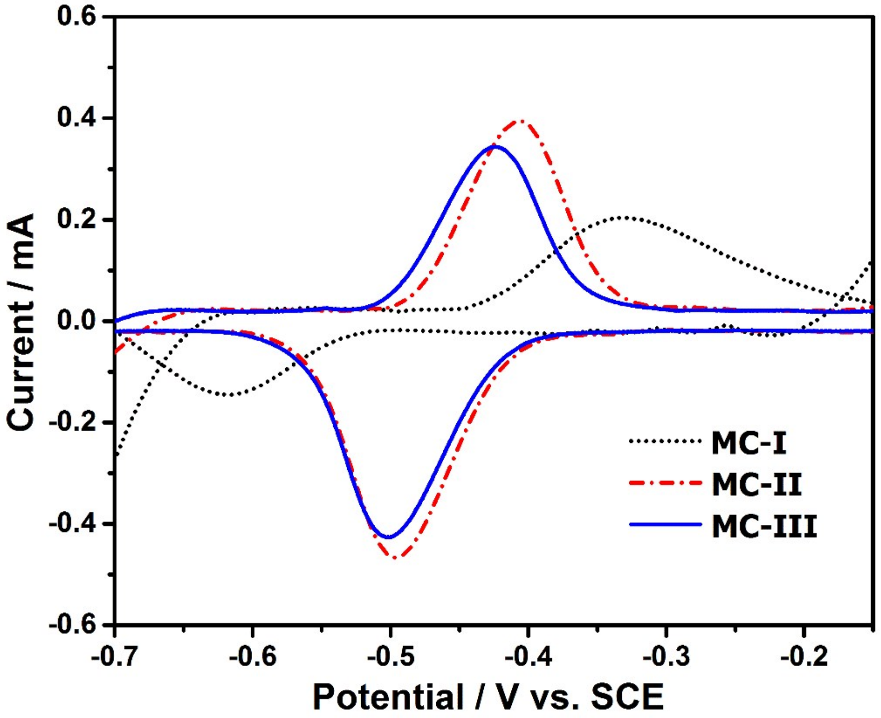

Figure S4. Baseline subtracted CVs of OMC anodes in anaerobic M9 buffer with *S. putrefaciens CN32* cell suspension. (Scanrate is 30 mV s^{-1})

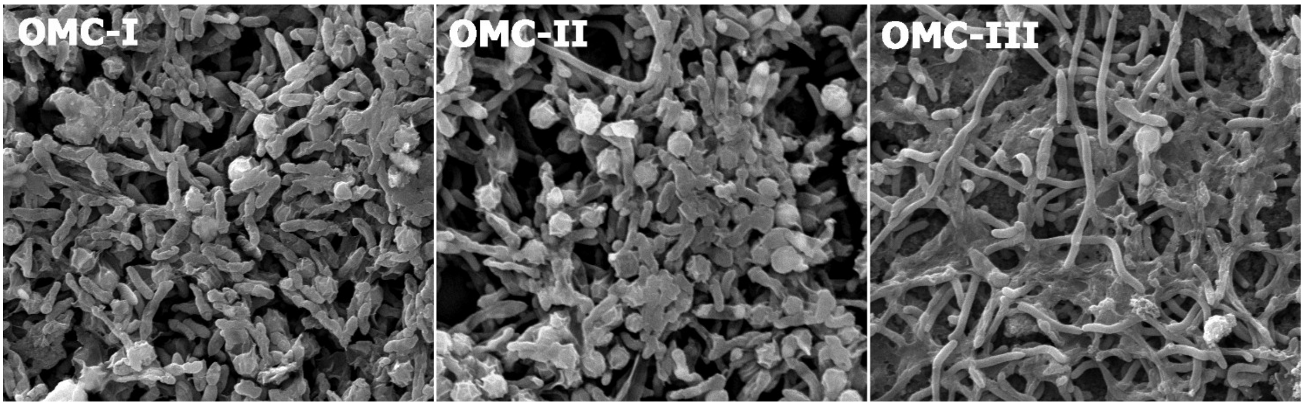

Figure S5. FESEM images of the biofilm adhered anodes after long term discharge.
